# l-Arginine in Mitochondrial Encephalopathy, Lactic Acidosis, and Stroke-like Episodes: A Systematic Review

**DOI:** 10.1212/WNL.0000000000200299

**Published:** 2022-06-07

**Authors:** Renae J. Stefanetti, Yi Shiau Ng, Linda Errington, Alasdair P. Blain, Robert McFarland, Gráinne S. Gorman

**Affiliations:** From the Wellcome Centre for Mitochondrial Research (R.J.S., Y.S.N., A.P.B., R.M., G.S.G.), Translational and Clinical Research Institute (R.J.S., Y.S.N., A.P.B., R.M., G.S.G.), and Faculty of Medical Sciences (R.J.S., Y.S.N., L.E., A.P.B., R.M., G.S.G.), Newcastle University; NIHR Newcastle BRC (R.J.S., Y.S.N., A.P.B., R.M., G.S.G.); and NHS Highly Specialised Service for Rare Mitochondrial Disorders (Y.S.N., R.M., G.S.G.), Newcastle Upon Tyne Hospitals NHS Foundation Trust, Newcastle Upon Tyne, UK.

## Abstract

**Background and Objectives:**

Stroke management in the context of primary mitochondrial disease is clinically challenging, and the best treatment options for patients with stroke-like episodes remain uncertain. We sought to perform a systematic review of the safety and efficacy of l-arginine use in the acute and prophylactic management of stroke-like episodes in patients with mitochondrial disease.

**Methods:**

The systematic review was registered in PROSPERO (CRD42020181230). We searched 6 databases from inception to January 15, 2021: MEDLINE, Embase, Scopus, Web of Science, CINAHL, and ClinicalTrials.gov. Original articles and registered trials available, in English, reporting l-arginine use in the acute or prophylactic management of stroke-like episodes in patients with genetically confirmed mitochondrial disease were eligible for inclusion. Data on safety and treatment response were extracted and summarized by multiple observers. Risk of bias was assessed by the methodologic quality of case reports, case series, and a risk-of-bias checklist for nonrandomized studies. Quality of evidence was synthesized with the Oxford Centre for Evidence-Based Medicine Levels of Evidence and Grade of Recommendations. The predetermined main outcome measures were clinical response to l-arginine treatment, adverse events, withdrawals, and deaths (on treatment and/or during follow-up), as defined by the author.

**Results:**

Thirty-seven articles met inclusion criteria (0 randomized controlled trials; 3 open-label; 1 retrospective cohort; 33 case reports/case series) (N = 91 patients; 86% m.3243A>G). In the case reports, 54% of patients reported a positive clinical response to acute l-arginine, of which 40% were concomitantly treated with antiepileptic drugs. Improved headache at 24 hours was the greatest reported benefit in response to IV l-arginine in the open-label trials (31 of 39, 79%). In 15 of 48 patients (31%) who positively responded to prophylactic l-arginine, antiepileptic drugs were either used (7 of 15) or unreported (8 of 15). Moderate adverse events were reported in the follow-up of both IV and oral l-arginine treatment, and 11 patients (12%) died during follow-up or while on prophylactic treatment.

**Discussion:**

The available evidence is of poor methodologic quality and classified as Level 5. IV and oral l-arginine confers no demonstrable clinical benefit in either the acute or prophylactic treatment of mitochondrial encephalopathy, lactic acidosis, and stroke-like episodes, with more robust controlled trials required to assess its efficacy and safety profile.

Mitochondrial diseases are the most common group of inherited neurometabolic diseases with a prevalence of 1 in 4,300. They are frequently multisystemic in nature and exhibit extensive heterogeneity of both genotype and phenotype. The clinical management of stroke-like episodes in the context of mitochondrial disease is especially challenging. These paroxysmal events define the mitochondrial encephalopathy, lactic acidosis, and stroke-like episodes (MELAS) syndrome and are considered manifestations of seizure activity that is often refractory/superrefractory to conventional anticonvulsive treatments.^1^

The exact mechanisms and pathophysiology of stroke-like episodes remain elusive with historically 2 leading hypotheses: microangiopathy vs neuronal hyperexcitability and cytopathy.^2^ Endothelial dysfunction, reduced nitric oxide (NO) synthesis rates,^3^ and low plasma concentrations of l-arginine^4^ in patients with MELAS have supported the argument for a therapeutic role for l-arginine (a potent NO donor) in mitochondrial stroke-like episodes. This has led to increasing advocacy of l-arginine in the treatment of MELAS,^5-8^ despite a paucity of supporting evidence. To close this gap in knowledge, we systematically investigated the efficacy and safety of l-arginine (and sought to undertake a meta-analysis) in individuals with reported stroke-like episodes and genetically confirmed mitochondrial disease.

## Methods

### Standard Protocol Approvals, Registrations, and Patient Consents

This systematic review was performed in accordance with Preferred Reporting Items for Systematic Reviews and Meta-Analyses guidelines (checklist available in eTable 1, links.lww.com/WNL/B836; checklist extensions available in eTables 2 and 3) and prospectively registered in PROSPERO International Prospective Register of Systematic Reviews (CRD42020181230). Ethics approval was not required for this systematic review because all data used were extracted from publications.

### Search Strategy

We searched MEDLINE, Embase, Scopus, Web of Science, and CINAHL from inception to January 15, 2021, with no language restrictions (see eAppendix 1, links.lww.com/WNL/B836). We performed backward citation searching, hand searching to manually screen the reference lists of included articles and related reviews, and searched ClinicalTrials.gov.

### Eligibility Criteria

The following inclusion criteria were applied: (1) author-defined mitochondrial stroke reported in the context of stroke-like episodes, encephalopathy, or seizures/epilepsy (MELAS) syndrome; (2) genetically confirmed mitochondrial disease; and (3) l-arginine treatment in the acute or prophylactic setting of stroke-like episodes management. Articles were eligible for inclusion when confirmation of stroke-like episodes was considered clinically relevant, with or without neuroimaging or changes on the EEG. No restrictions were placed on study design, outcome measures, or date of publication to be as comprehensive as possible. Data from unpublished abstracts or conference proceedings were excluded, in addition to articles reporting response to l-arginine in the same patient cohort in which no new information on treatment response is provided.

### Study Selection

Two reviewers (R.J.S. and G.S.G.) independently screened all records by titles and abstracts for eligibility, and 3 authors (R.J.S., G.S.G., Y.S.N.) assessed the full texts of potentially eligible studies to determine eligibility for final inclusion (eTable 4, links.lww.com/WNL/B836). Conflicts on inclusion of articles were resolved by consensus through discussion.

### Data Extraction

Data extraction from included articles was performed independently by 2 authors (R.J.S. and Y.S.N.) The data extracted included article study design, patient demographics, genetic diagnosis, clinical presentation of stroke-like episodes, sample size, additional treatments, interventional details (dose, route, time of administration from symptom onset, treatment duration, length of follow-up), clinical outcome response (concomitant antiepileptic drug [AED] treatment), adverse events (AEs), trial withdrawals, and deaths on treatment and during follow-up.

### Risk of Bias

Risk of bias was assessed by 2 authors independently (R.J.S. and Y.S.N.). We used a recently developed tool for evaluating the methodologic quality of individual case reports and case series.^e1^ The overall quality appraisal for each case report or case series was classified according to the number of questions satisfied across any domains of ascertainment, causality, and reporting; ≥3 questions = good quality, 2 questions = poor quality, 1 or 0 questions = very poor quality. Risk of bias for all other articles was assessed with a checklist for nonrandomized studies.^e2^ Articles were deemed to have an overall high risk of bias if their analyses did not adjust for (or report) the influence of confounders as deemed by the investigators (i.e., use of AEDs) and if participant withdrawals were likely to introduce bias. The quality of evidence for individual studies was rated and synthesized with the Oxford Centre for Evidence-Based Medicine's (OCEBM’s) Levels of Evidence (March 2009) and Grades of Recommendation.^e3^ In relation to therapy, evidence can range from Level 1 (systematic review [with homogeneity] of randomized controlled trials) to Level 5 (expert opinion without explicit critical appraisal or based on physiology). In between (in descending strength of evidence), major levels include Level 2(a), a systematic review of cohort studies; Level 2(b), individual cohort studies (or lower-quality randomized controlled trials); Level 3(a), systematic reviews (with homogeneity) of case-control studies; Level 3(b), individual case-control studies; and Level 4, case-series (and poor-quality cohort and case-control studies). A Grade of Recommendation will thereafter be adapted, ranging from A (consistent Level 1 studies) to D (Level 5 evidence).

### Statistical Analysis

The methodologic quality of the included studies was limited, consisting predominantly of case reports or case series. Compatible results were pooled. Heterogeneity of interventional parameters and outcome reporting precluded a meta-analysis. Incomplete datasets and the degree of covariate imbalance precluded interpretation in a multinomial logit regression setting.

### Data Availability

All data relating to this systematic review (articles reviewed, statistical analysis, raw data tabulations) will be available on request by any qualified investigator. Supplemental material is available in a publicly accessible data repository (Figshare; doi.org/10.25405/data.ncl.16514172).

## Results

**Figure 1:**
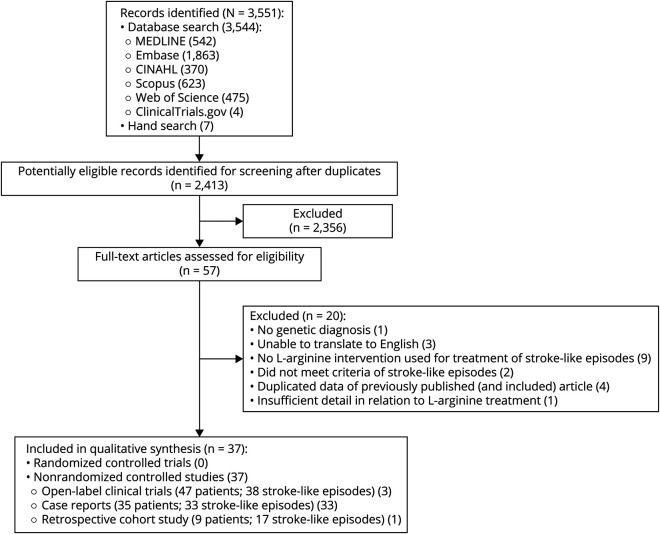
PRISMA Flow Diagram PRISMA = Preferred Reporting Items for Systematic Reviews and Meta-Analyses.

The screening and selection of articles are described in Figure 1. Of 3,551 articles, 37 articles including 91 participants met the selection criteria, with the majority of individuals harboring the m.3243A>G pathogenic variant in the *MT-TL1* gene (86%) (summary characteristics detailed in eTables 5 and 6, links.lww.com/WNL/B836).

**Figure 2:**
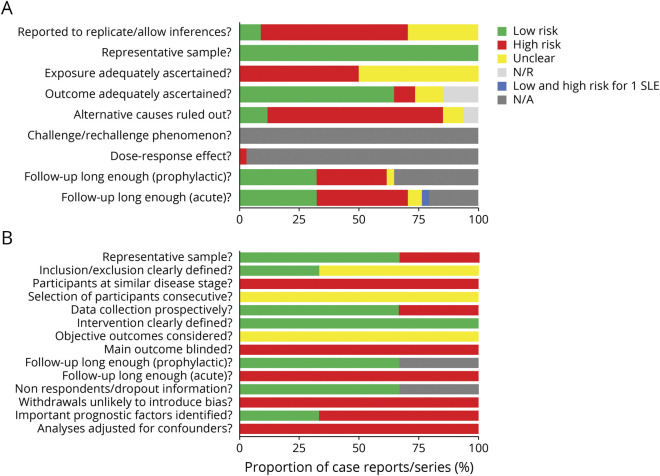
Quality Appraisal and Risk of Bias Risk of bias for case reports and case series (n = 34), evaluated with the tool developed by Murad et al.^e1^ Color denotes the risk of bias (see legend), defined by meeting the criteria standards for each domain (on the y-axis). Each article was individually appraised. The x-axis represents the combined proportion of case reports/case series. eTable 7, links.lww.com/WNL/B836, gives the individual article appraisal. (B) Quality appraisal and risk of bias for cohort and open-label trials (n=3). Risk of bias for all other study design was evaluated via a risk-of-bias checklist for nonrandomized studies developed by Brazzelli et al.^e2^ Color denotes the risk of bias (see legend), defined by meeting the criteria standards for each domain (on the y-axis). Each article was individually appraised. The x-axis represents the combined proportion of case reports/case series. eTable 8 gives the individual article appraisal. N/A = not applicable; N/R = not reported; SLE = stroke-like episode.

Of the 37 articles, none were randomized, controlled interventional studies; 3 were open-label trials^4,9,10^ (placebo controlled in 2 trials)^4,10^; 1 was a retrospective cohort study^9^; and the remaining articles consisted of case reports and case series (89%). Study quality was rated as poor or very poor in 30 of 34 (88%) of case reports/case series (Figure 2A and eTable 7, links.lww.com/WNL/B836), with domains of causality, ascertainment, and reporting deemed high risk of bias. Three open-label trials and the single-cohort study were also classified as having an overall high risk of bias, with confounders similarly unaccounted for, i.e., AED use (Figure 2B and eTable 8). Of the 37 included articles, level of evidence according to the OCEBM hierarchy included Grade 5 (n = 33), Class 4 (n = 1), and Class 3 (n = 3) (eTable 8).

### Clinical Presentation

The mean age of all patients was 26.2 years (SD 18.4 years) at the time of l-arginine treatment. There were considerable differences in the mean age (95% CI), and this varied across study designs, while the proportion of women was consistently slightly higher (albeit not reported in the cohort study) (Table 1).

**Table 1:**
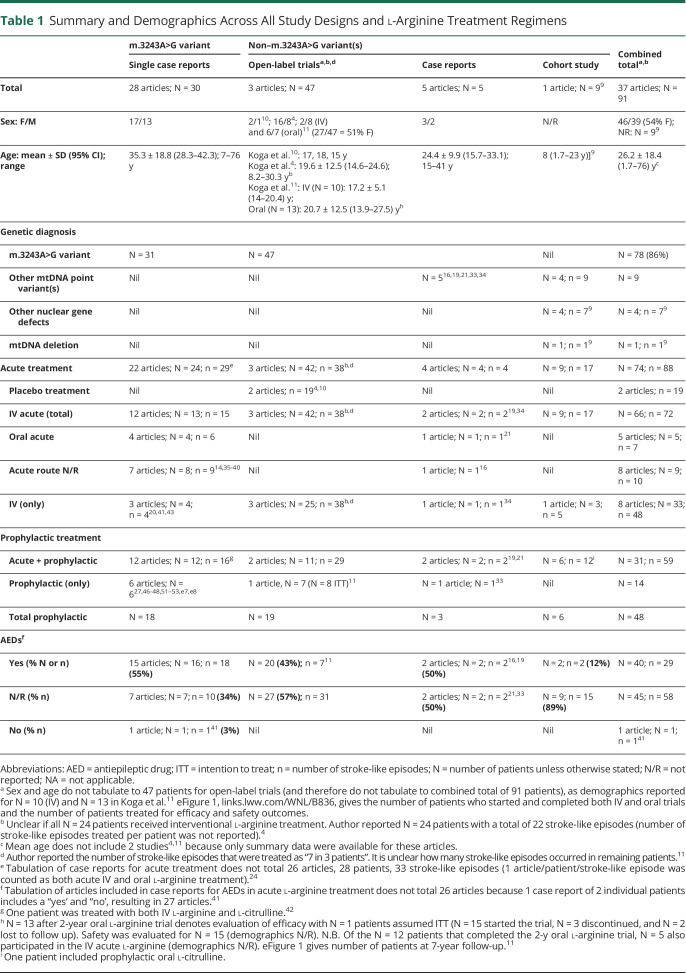
Summary and Demographics Across All Study Designs and l-Arginine Treatment Regimens

Of the case reports, elevated blood lactate (>2.2 mM, range 2.2–9.9 mM) was the most frequent laboratory finding associated with stroke-like episodes (83%; Table 2). Seizures were the most frequent presenting feature of stroke-like episodes (73%; Table 2). In contrast, patients in the open-label trials who presented with headache, nausea/vomiting, positive visual phenomena, or visual field loss were not reported to present with seizures despite AED use^11^ (Table 2).

**Table 2:**
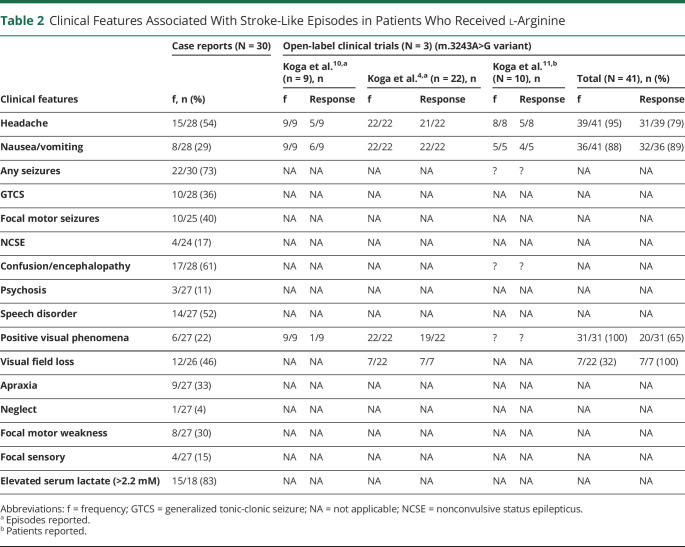
Clinical Features Associated With Stroke-Like Episodes in Patients Who Received l-Arginine

### Treatment

Ninety-one patients were treated with l-arginine acutely, chronically, or a with combination of both (85% m.3243A>G). l-Arginine was used as an acute treatment in 66 patients (72 stroke-like episodes); IV l-arginine was administered to 33 patients (48 stroke-like episodes), while 48 patients received oral l-arginine as a prophylactic treatment (Table 1).

In the case reports, 18 of 28 patients (64%) who received acute l-arginine also received AEDs, vitamins (n = 16), or other pharmacologic agents (n = 16).

The dosage for the IV l-arginine in the open-label trials was consistently a single dose of 0.5 g/kg, although in the Koga et al.^11^ study, repeated dosing of 0.5 g/kg at 2 hours after initial infusion was administered if symptoms did not improve. Conversely, there was considerable variability in the IV dose (and regimen in the cohort study [0.2–1.5 g/kg/d]^9^ and the case reports (eTable 5, links.lww.com/WNL/B836). Time of administration from symptom onset also varied and was not consistently reported (eTable 5); for example, in the open-label trials, this varied from 1^4^ to 6^11^ hours from symptom onset.

### Efficacy: Acute l-Arginine

Of the 28 patients treated acutely with l-arginine in the case reports/case series, 54% of patients (15 of 28) and 55% (18 of 33) of stroke-like episode events were reported to respond positively to therapy (Table 3). However, 40% of the patients (6 of 15) who had an improvement were also treated concomitantly with AEDs (eTable 5, links.lww.com/WNL/B836). Six patients deteriorated clinically, with worsening seizures, despite acute treatment with l-arginine,^12-17^ requiring escalation of AED therapy, including admission to the intensive care unit.^16^ Radiologic changes after l-arginine treatment were reported as improvements in magnetic resonance spectroscopy parameters and partial or full resolution of stroke-like lesions on brain MRI (Table 3).

**Table 3:**
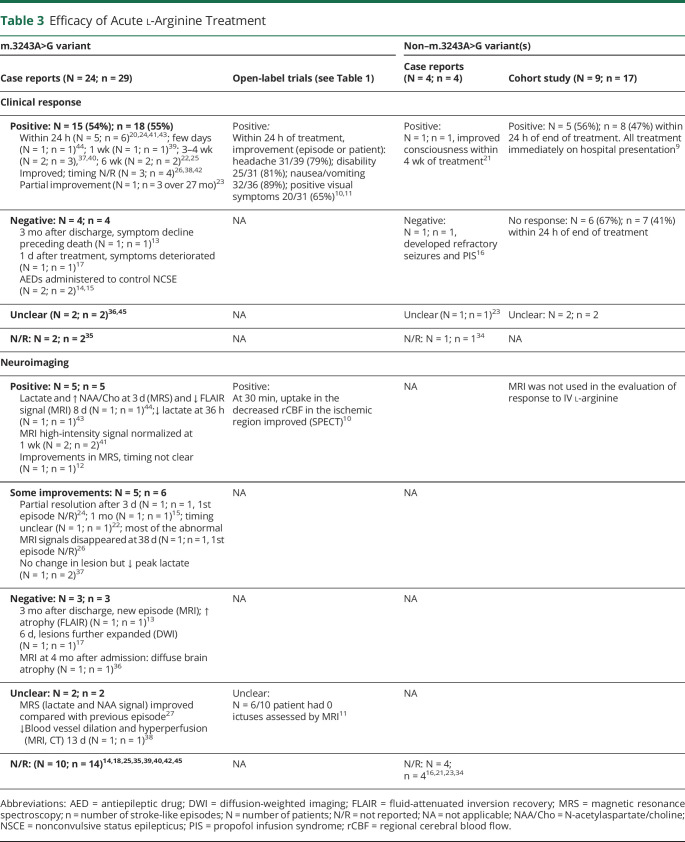
Efficacy of Acute l-Arginine Treatment

Of the open-label trials, symptomatic improvement in headache (79%) showed highest reported benefit in response to acute l-arginine treatment within 24 hours (eTable 6, links.lww.com/WNL/B836), with other reported improvements in vomiting/nausea (89%), clinical disability (81%), and teichopsia (65%).^4,10,18^ However, it was unclear whether these improvements were sustained beyond 24 hours. All patients reported by Koga et al.^18^ had been prescribed at least 1 AED. However, we were unable to ascertain the temporal relationship between instigation of AED and dosing with IV l-arginine.^18^

In the cohort study, conducted by Ganetzky and Falk^9^ of the 9 patients with a total of 17 stroke-like episodes treated with IV l-arginine, 3 patients were reported to have responded, 4 patients had no response, and 2 patients demonstrated variable response yet had received concomitant AED therapy.^9^

### Prophylactic l-Arginine

A total of 48 patients across the study designs were treated with oral l-arginine (Table 4), although the dose and duration were variable between studies (eTable 5, links.lww.com/WNL/B836). Fifteen patients (31%) had a positive response, with the severity or frequency of stroke-like episodes reduced. While AED use was not reported in 2 of the 3 open-label trials,^4,10^ AEDs were prescribed simultaneously in 7 of 9 patients who responded in the case reports. Stroke-like episodes recurred in 54% of patients (26 of 58) who received prophylactic oral l-arginine, while treatment response was not reported in 15% (n = 17) of articles.

**Table 4:**
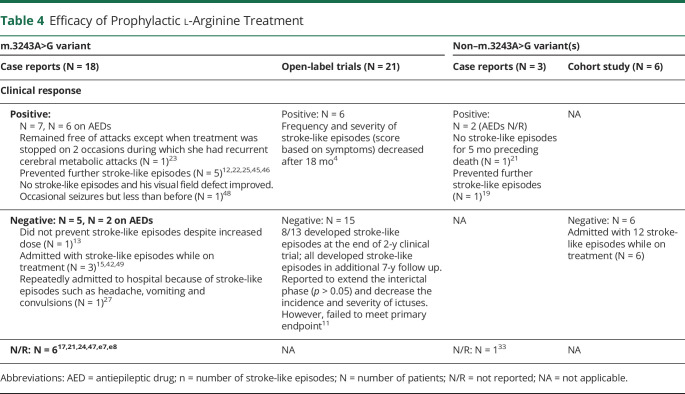
Efficacy of Prophylactic l-Arginine Treatment

### Safety of IV l-Arginine

In response to IV l-arginine, 5 articles (3 case reports/case series,^12,19,20^ 1 cohort,^9^ 1 open label^10^) specifically reported no associated AEs related to therapy. While the reporting of AEs was absent in all remaining case reports, AEs were reported in 2 open-label trials. Moderate AEs reported by Koga et al.^11^ included fever (n = 5), decreased hematocrit (n = 3), hemoglobinuria (n = 3), bleeding at the injection site (n = 1), and epilepsy (n = 1) (Table 5). Deterioration in clinical status also occurred in 1 patient, which was attributed by the authors to the patient being prone to seizures. Two patients also developed a headache when l-arginine was infused too rapidly.^4^

**Table 5:**
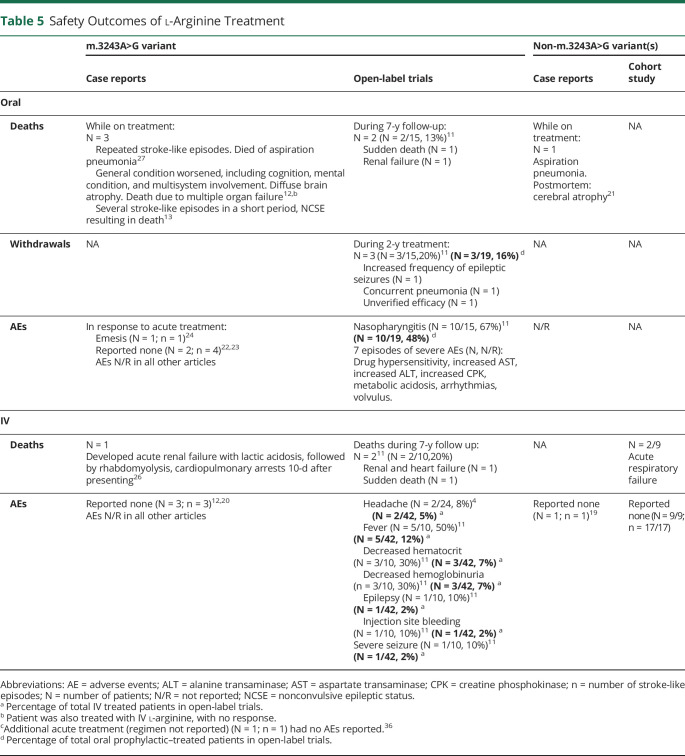
Safety Outcomes of l-Arginine Treatment

### Oral l-Arginine

Nasopharyngitis was the most common AE^11^ in patients on prophylactic oral l-arginine (n = 10). Seven episodes of what the authors reported as severe AEs were noted in patients on prophylactic oral l-arginine, including drug hypersensitivity; increased aspartate transaminase, alanine transaminase, and creatine phosphokinase; metabolic acidosis; arrhythmias; and volvulus. All AEs resolved on discontinuation of oral l-arginine. Three patients discontinued oral treatment in a 2-year open-label trial due to increased frequency of seizures (n = 1), concurrent pneumonia (n = 1), and unverified efficacy (n = 1). Of the 5 patients treated acutely with oral l-arginine,^21-25^ emesis occurred in a single patient.^24^

### Mortality (IV and Oral l-Arginine)

Eleven (12%) of all 91 patients treated with l-arginine (both oral and IV) died during follow-up or while on prophylactic (oral) treatment (Table 5). Five patients who received IV l-arginine died during the acute admission with stroke-like episodes (renal failure [n = 1],^26^ acute respiratory failure [n = 2^9^]) or during follow-up (combined renal and heart failure [n = 1], sudden death [n = 1^11^]). Six patients treated with prophylactic oral l-arginine died, with cause of death attributed to aspiration pneumonia (n = 2),^21,27^ multiple organ failure (n = 1),^12^ nonconvulsive status epilepticus (n = 1),^11^ sudden death (n = 1),^11^ and combined renal and heart failure (n = 1).^11^

## Discussion

This systematic review provides a comprehensive summary of the evidence for the efficacy and safety of the use of l-arginine in patients with stroke-like episodes in the context of genetically defined MELAS syndrome. We found that no randomized clinical trials have been undertaken and that the studies included encompass significant methodologic limitations. The certainty of evidence was classified as D: Level 5 Grade of Recommendation, that is, expert opinion without explicit critical appraisal, troublingly inconsistent or inconclusive.

As described in the original reports of MELAS,^28^ patients' neurologic deficits may improve after acute stroke-like episodes; hence, in the absence of placebo-controlled randomized clinical trials, it is impossible to be certain that improvements after stroke-like episodes are due to l-arginine as opposed to the natural evolution of a stroke-like episode. In the acute treatment of mitochondrial stroke-like episodes, individual data based on case reports/case series and a single retrospective cohort study^9^ showed that about half of all patients had a positive clinical response as deemed by the authors. However, the concomitant use of AEDs or other pharmacologic treatments severely compromises the attribution of clinical improvement to l-arginine alone. In the open-label trials, while a symptomatic improvement in headache, clinical disability, vomiting/nausea, and teichopsia was found, the response was not reported beyond 24 hours.^4,10,11^ Furthermore, while these particular clinical features often accompany stroke-like episodes, they are also observed in migraine, not an uncommon clinical complication of m.3243A>G–related mitochondrial disease. In the prophylactic use of l-arginine, there was no clear evidence that l-arginine reduces the duration or prevents the recurrence of stroke-like episodes (Table 4). The salient features of MELAS-associated headache, forming a major component of many trial inclusion criteria and outcomes, were poorly defined. The rapid resolution of symptoms and arbitrary scales used raise concern that some episodes could simply have been migraine.

Our findings challenge a recent consensus statement and several clinical practice guidelines that endorse the use of l-arginine.^5-8,29-31^ In the acute setting of stroke-like episodes associated with MELAS, the urgent administration of IV l-arginine is recommended as a continuous infusion for anywhere between 1 and 5 days.^5,8,30^ For prophylactic use, it is recommended that use of daily oral l-arginine supplementation be considered to prevent stroke-like episodes.^8,30^ This systematic review of the literature cannot find any evidence beyond Level 5 to support these recommendations. Moreover, these expert opinion and clinical recommendation fail to mention use of AEDs,^5-8,29,30^ and very few^5,29^ comment on the potential AEs associated with high-dose l-arginine.

Variation in trial design, definition of stroke-like episodes, timing of l-arginine administration, and evaluation of clinical response and period of follow up were commonplace (eTables 5 and 6, links.lww.com/WNL/B836). Limitations in our understanding of the mechanisms involved in MELAS syndrome may be reflected in the controversies surrounding the definition of a stroke-like episode. In the open-label trials, the definition of a stroke-like episode relied primarily on clinical symptoms alone^4,9,10^ (e.g., headaches, nausea, and vomiting) without supporting evidence from neuroimaging or EEG. On the other hand, brainstem dysfunction in the context of Leigh syndrome crisis was also regarded as stroke-like episodes in another study.^9^ Neither of these definitions would fulfill a recent consensus-based statement defining stroke-like episodes^1^ or historical diagnostic criteria.^28,32^ Acute l-arginine treatment was administered within 1 hour^4,10^ to 6 hours^11^ from symptom onset in open-label trials, while in the case reports, the time interval between the onset of stroke-like episode and l-arginine treatment often ranged from days to weeks. Moreover, the response to acute (IV) l-arginine was determined on the basis of the resolution of clinical symptoms alone within 24 hours in the open-label trials.^4,10,11^ In contrast, the majority of case reports/case series reported changes in clinical presentation and neuroimaging appearance (with or without EEG). The follow-up period was up to 24 and 48 hours in the open-label studies^4,10,11^ and retrospective cohort study,^9^ respectively, compared to weeks and months in the case report/case series (eTable 5).

The presence or absence of AEs was not consistently reported, and when serious AEs were documented, their causal relationship with l-arginine was invariably disputed. Clinicians and patients with MELAS syndrome should be made aware of the potential serious complications associated with high doses, long-term use, and limited monitoring of l-arginine, which include hyperkalemia, profound metabolic acidosis requiring dialysis, and, in some instances, sudden death.^e4-e6^ This is particularly pertinent in those individuals with significant cardiac or renal impairment or persistent lactic acidemia.

The limitations of this systematic review are related to the small body of literature available and the potential impact of the genetic and clinical heterogeneity in mitochondrial disease contributing to the l-arginine treatment response. Additional methodologic limitations relate to the inclusion of nonrandomized studies, predominantly lacking a comparison group, and the lack of standardized outcome measures across studies used to assess treatment response. Most studies available were case reports with a high risk of bias, including insufficient reporting and ascertainment. Studies included were difficult to compare due to differences in interventional parameters (dose, regimen, length of treatment, time of administration, simultaneous treatment with AEDs) and assessment of response to treatment (i.e., outcome measures used, methodology, timing of response). In this respect, a lack of heterogeneity across studies precluded a meta-analysis.

Despite its limitations, this systematic review had several strengths such as a prespecified protocol, a comprehensive search strategy, an absence of article eligibility restrictions to allow greater inclusion, and standardized assessment of risk of bias, culminating in a comprehensive and objective summary of the evidence that could be useful in guiding clinical practice and future research.

Our findings demonstrate that l-arginine has a very limited efficacy in the acute and prophylactic treatment of stroke-like-episodes (Level V evidence). The risk of AEs could not be determined with certainty from the current published data. These findings highlight that methodologically robust clinical trials are imperative to address the remaining uncertainty relating to the treatment of l-arginine in patients with MELAS syndrome. Because stoke-like episodes are increasingly recognized as evolving brain syndromes driven by seizure activity,^1,50^ seizure treatments, including infusions of AEDs or use of anesthesia agents, should be prioritized in these patients. While there are potential cultural differences in shared decision-making for medical practice in rare diseases globally, we would encourage mitochondrial experts to share this information about l-arginine with patients and colleagues to allow them to make an informed decision.

## Glossary

AE: adverse event
AED: antiepileptic drug
MELAS: mitochondrial encephalopathy, lactic acidosis, and stroke-like episodes
NO: nitric oxide
OCEBM: Oxford Centre for Evidence-Based Medicine
